# Exploring the Relationship Between the Size and Diverticular Number of Carpal Gland in Four Pig (*Sus Scrofa*) Populations

**DOI:** 10.3390/ani14223231

**Published:** 2024-11-11

**Authors:** Dengshuai Cui, Naibiao Yu, Sanya Xiong, Ruiqiu He, Shijun Xiao, Longyun Li, Yuanmei Guo

**Affiliations:** National Key Laboratory of Pig Genetic Improvement and Germplasm Innovation, Jiangxi Agricultural University, Nanchang 330045, China; yumike18720547422@gmail.com (N.Y.);

**Keywords:** pig, carpal gland, diverticular number, weight, volume, correlation coefficient

## Abstract

The carpal gland is an important exocrine gland in pigs, functioning mainly through diverticula that release strongly odorous secretions. The size of the carpal gland and the number of diverticula may be related to its function. To measure its size, the gland must be dissected from the foreleg, and this procedure is time-consuming and cumbersome. The diverticulum is the outlet of the carpal gland, and its number correlates with the carpal gland size, but the correlation coefficient between them is still unknown. To explore the relationship between the size and diverticular number of the carpal gland, 788 pigs from four populations were used in this study. The length, width, thickness, weight and volume of the carpal gland were measured, together with its diverticular number. The phenotypic correlation coefficient between each pair of the recorded traits was calculated, and a two-factor analysis of variance was used to test whether sex and population have effects on the phenotypes. The carpal gland is a flat small stick with a length of 1.8–8.6 cm, width of 0.6–2.5 cm, and thickness of 0.2–0.8 cm, and its specific gravity is 0.51–1.36. Each carpal gland has 0 to 10 diverticula, and 82.3% carpal glands have 2–6 diverticula. The diverticular number correlates positively with the weight (*r* = 0.57 and *r_s_* = 0.58, *p* < 2.2 × 10^−16^) and volume (*r* = 0.55 and *r_s_* = 0.56, *p* < 2.2 × 10^−16^). Population and sex only explain 20% of the phenotypic variance, suggesting that the 80% of carpal gland influences remain unknown. In conclusion, the diverticular number can be used as a proxy for the weightand volume of the carpal gland, because it is easy to count and has a significant positive correlation with the size of thecarpal gland.

## Simple Summary

The carpal gland is an important exocrine gland in pigs, and its size may be related to its function, but measuring its size is time-consuming and cumbersome. The diverticulum is the outlet of the carpal gland, and its number is easy to count. The diverticular number positively correlates with the carpal gland size, but the correlation coefficient between them is still unknown. To estimate the correlation coefficient between the size and diverticular number of the carpal gland, the weight and volume of the carpal gland were measured, together with its diverticular number. The diverticular number correlates positively with the weight (*r* = 0.57 and *r_s_* = 0.58, *p* < 2.2 × 10^−16^) and volume (*r* = 0.55 and *r_s_* = 0.56, *p* < 2.2 × 10^−16^). Therefore, the diverticular number can be used as a proxy for the weight and volume of the carpal gland.

## 1. Introduction

The carpal gland is the large, irregular and centrally distributed cutaneous gland in pigs, and it is located on the inner side of the carpal joint of the forelimbs in both sexes [1,2]. The gland plays an essential role in protecting the integrity of the pig’s skin against external viral invasions [3,4,5], communication between individuals, and population reproduction [1,2,5,6,7,8,9,10] by secreting strongly odorous secretions [11,12]. In recent years, with increasing numbers of feral pigs, they have become an economic pest, posing a serious threat to crops, buildings and ecosystems in many areas (e.g., destruction of seeds, vegetation and native wildlife through food foraging), and acting as a reservoir for diseases that can be transmitted to native mammals [9,10,13]. The strongly odorous secretion of the carpal gland induces avoidance behavior in wild boars and can be used as a repellent to repel wild boars from farms or as a trapping agent to control wild boar populations, thereby reducing wild boar destruction [9,14]. Notably, our previous study also found that the carpal gland diverticular number was significantly correlated with important meat quality traits such as 24 h pH, 24 h meat color score, 24 h marbling, fat content, moisture content, sodium salt content, and saturated fatty acid content [15]. Therefore, the study of the size, number of diverticula and function of the carpal gland is valuable in reducing destruction of crops, buildings, and ecosystems in feral boars, as well as in pig production.

The functional strength of carpal gland is positively correlated with its size. To measure itssize, the carpal gland is dissected from the forelimbs after the pig is slaughtered [9,16]. This process is time-consuming and costly; furthermore, the pig is unavailable for further studies after having been slaughtered. For efficient phenotyping, we developed a method to measure the carpal gland size by counting its diverticular number in our previous study [15]. The diverticular number of the carpal gland can be counted after the pig was slaughtered and depilated, without dissecting the carpal gland from the forelimbs. Although the size and diverticular number of the carpal gland are strongly and positively correlated with each other, the correlation coefficient between them is still unknown.

In this study, the size and diverticular number of the carpal gland were recorded in four populations, namely, Canadian Large White, French Large White, Landrace and Duroc. The correlation coefficient between the size and diverticular number of the carpal gland was estimated. If their correlation is strongly and highly significant, the diverticular number can be used as a proxy for the size of the carpal gland, because the former is easy to record.

## 2. Materials and Methods

### 2.1. Ethics Approval

The experimental pigs were treated according to the guidelines for the care and use of experimental animals issued by the Ministry of Agriculture and Rural Affairs of China, and The Animal Care and Use Committee of Jiangxi Agricultural University approved this study.

### 2.2. Experimental Animals

A total of 788 pigs (330 barrows and 458 gilts) from four populations were utilized in this study, including 309 Canadian Large White pigs, 250 French Large White pigs, 130 Landrace pigs and 99 Duroc pigs. All of the pigs were raised according to the normal nutritional standard, and they were given access to the feed and fresh water ad libitum. The pigs were slaughtered at the same commercial abattoir when their body weights were about 110 kg.

### 2.3. Phenotyping

After the pigs were slaughtered and depilated, the diverticular number of the carpal gland in each foreleg was recorded. To measure the size of the carpal gland, the whole carpal gland was dissected from the right foreleg. When the other tissues were completely stripped away, the carpal glands were weighed using a small electronic scale (Dongguan, China) with a precision of 0.01 g (Figure S1A). The length, width and thickness of the carpal gland were measured using a ruler with a precision of 0.1 cm (Figure S1B), and the color of the carpal gland was scored from 1 (light yellow) to 3 (dark yellow). Moreover, the volume of the carpal gland was measured with the two methods: one is a drainage method (*V*), and the other is the regression volume (*V*_reg_). A 10 mL volumetric cylinder with the precision of 0.1 mL was partly filled with water, and the volume of water was read as *V*_1_. Then, the carpal gland was dipped into the water completely, and the total volume of water and carpal gland was read as *V*_2_. The drainage volume (*V*) is equal to *V*_2_ − *V*_1_. In each population, a regression equation of *V* to carpal gland weight was fitted, and *V*_reg_ of each individual was the predicting volume of the regression equation given its carpal gland weight.

### 2.4. Statistical Analysis

All statistical analyses were performed in R (R 4.3.2). The mean and standard deviation of each trait were calculated in each population using the *mean* and *sd* functions, respectively. The phenotypic correlation coefficient between each pair of the recorded traits was calculated using the *cor.test* function. For the diverticular numbers, a Wilcoxon rank sum test and a Kruskal–Wallis test were performed to check the effects of sex and populations, and the two nonparametric tests were carried out using the *wilcox.test* and *kruskal.test* functions, respectively. For the other traits, a two-factor analysis of variance was employed to test the effects of sex and populations using the *lm* and *Anova* (in the car package) functions. Least square means (LSMs) were estimated using the *emmeans* function, and the Tukey method in the *paris* function was applied to address the multiple-comparison problem in the emmeans package (v 1.8.9). A paired two-sample *t* test was utilized to check the difference in the diverticular numbers between the left and right forelegs. Moreover, the variancePartition [17] package was employed to quantify the variation in carpal gland traits attributable to variation in different factors such as population, sex, and so on. The data were visualized using the *plot*, *pairs*, *hist*, *plotPercentBars*, *plotVarPart* and *corrplot* functions in the corrplot (v 0.92) and variancePartition (v 1.33.14) packages.

## 3. Results

### 3.1. Simple Descriptive Statistics of the Carpal-Gland-Related Traits

Table 1 lists the simple statistics of the diverticular number and size of carpal glands for each population. In the four populations, the average diverticular number of the carpal gland was less than 5 on each foreleg, and less than 10 on both forelegs. The carpal gland looked like a flat small stick with a length of 1.8–8.6 cm, width of 0.6–2.5 cm, and thickness of 0.2–0.8 cm. Its weight and volume were 0.15–7.16 g and 0.2–7.5 cm^3^, respectively.

### 3.2. Phenotypic Correlation Coefficients

The Pearson (*r*) and Spearman (*r_s_*) phenotypic correlation coefficient is shown in Table 2. The diverticular numbers on both forelegs were significantly correlated (|*r*| ≥ 0.11, *p* ≤ 0.0023; |*r_s_*| ≥ 0.11, *p* ≤ 0.0025) with the other traits, and they were also significantly and strongly correlated (*r* ≥ 0.76, *p* < 0.0001; *r_s_* ≥ 0.74, *p* ≤ 0.0001) with each other. For the carpal gland on the right foreleg, its diverticular number was positive and highly significant (*p* < 0.0001) and strong correlated with its weight (*r* = 0.57, *r_s_* = 0.58), length (*r* = 0.68, *r_s_* = 0.65) and volume (*r* = 0.55, *r_s_* = 0.55). The difference between the diverticular numbers on the left and right forelegs did not significantly correlate with other traits except the weight, length and volume of the right carpal gland. The specific gravity correlated with other traits weakly, and the correlation coefficients were |*r*| ≤ 0.15 and |*r_s_*| ≤ 0.16. The remaining traits were in significant correlation with each other except between length and thickness, for which the Pearson correlation coefficient was not significant (*r* = 0.07, *p* = 0.06), but the Spearman correlation coefficient was significant (*r_s_* = 0.08, *p* = 0.03).

### 3.3. Effects of Population on Carpal Gland Traits

Both the parametric and nonparametric tests yielded the same results: population had a significant effect on all recorded traits except the difference in diverticular numbers between the forelegs (Table 3). Duroc and Landrace had more diverticular numbers than Canadian Large White, and there was no significant difference among other populations. The carpal gland weights were significantly different from each other in the four populations. Landrace had the heaviest carpal gland, followed by Duroc, French Large White and Canadian Large White. Landrace also had the longer carpal gland than the other three populations, and the carpal gland length showed no significant difference among the three populations. Landrace had the widest and largest carpal gland, Duroc and French Large White followed, and Canadian Large White had the narrowest and smallest carpal gland. Canadian Large White had a thicker carpal gland than Landrace, and there was no difference among other populations.

### 3.4. Effects of Sex on Carpal Gland Traits

The effects of sex on carpal gland traits are listed in Table 4. The least square means of all traits were larger in males than in females, but the differences were not significant except for the volume of carpal gland (*p* = 0.02). The Wilcoxon rank sum tests did not correct the effect of population, and the results showed that the weight, length and volume of the carpal gland were significantly (*p* ≤ 0.04) different between sexes.

### 3.5. Effects of Legs on Carpal Gland Traits

The diverticular numbers showed no significant difference between the forelegs in each (*p* ≥ 0.32) and all (*p* = 0.68) of the four populations, and the diverticular number was symmetric in the four populations.

### 3.6. Phenotypic Variances Explained by Each Factor

**Figure 1:**
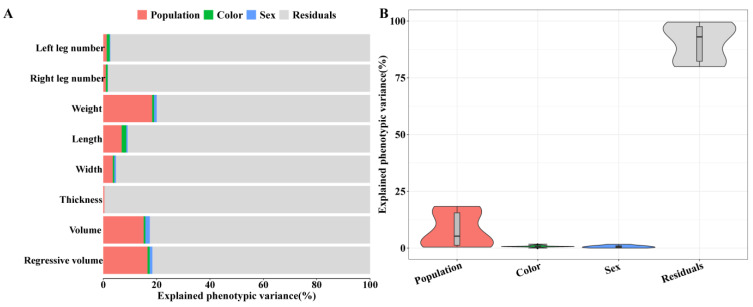
The percent phenotypic variances explained by population, sex, color and residual. (**A**) Histograms of percent phenotypic variances for each trait explained by population, sex, color and residual. (**B**) Violin plots of percent phenotypic variances for all traits explained by population, sex, color and residual.

The phenotypic variance of each recorded trait explained by population, sex, and carpal gland color are shown in Figure 1 and Table S1. Population had a significant effect on most carpal-gland-related traits, and it explained 18.33%, 15.16% and 16.61% phenotypic variances of the weight, volume and regressive volume of the carpal gland, respectively. Sex also had a significant effect on most traits, and it explained 0.99%, 1.63% and 1.03% phenotypic variance of the weight, volume and regressive volume, respectively. Color had the same order of magnitude of phenotypic variance as sex, suggesting that it also had a significant effect on some traits. At least 79.95% phenotypic variance was unexplained, indicating that some unknown factors needed to be identified.

### 3.7. Distributions of Carpal Gland Diverticular Numbers

The distributions of diverticular numbers in the four populations were showing in Figure S2. Most French Large White pigs had 4 diverticula on each foreleg, with 29.32% and 31.73% pigs having 4 diverticula in the left and right forelegs, respectively. In Canadian Large White, most pigs had 4 diverticula on their left forelegs (25%) and 3 on their right forelegs (22.76%). Most Duroc pigs, accounting for 25.74% and 19.80%, had 5 diverticula on left and right forelegs, respectively. Most Landrace pigs had 4 diverticula on the right foreleg, accounting for 33.08%, and had 5 on the left foreleg, accounting for 25%. For the joint population, the diverticula in each foreleg were in a range from 0 to 10, and more than 82.3% pigs had 2–6 diverticula on each foreleg.

### 3.8. Relationship Between the Diverticular Number and Weight

**Figure 2:**
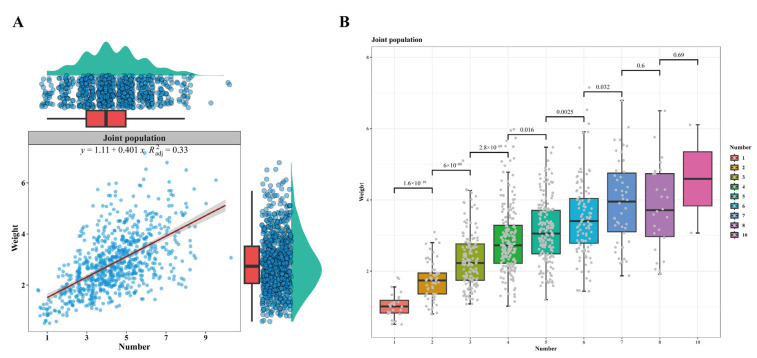
Scatter (**A**) and box (**B**) plots between the diverticular number and weight of the right carpal gland. The red line is the fitted straight line between the diverticular number and weight of the carpal gland.

Figure 2 shows the scatter and box plots of the carpal gland weight against the diverticular number in the joint population. There was a positive correlation between the weight and diverticular number of the carpal gland on the right foreleg, and the intercept and regression coefficient of the former regressing on the latter were 0.40 ± 0.020 and 1.11 ± 0.095 (Figure 2A), respectively. All of the average weights among the diverticular numbers were significant (*p* < 0.05) except for three pairs (Figure 2B), which were between 7 and 8 (*p* = 0.60), between 8 and 10 (*p* = 0.69), and between 7 and 10 (*p* = 0.74). Furthermore, we performed the same analysis in each of the four populations, and the results were consistent (Figure S3).

### 3.9. Relationship Between the Volume and Weight

**Figure 3:**
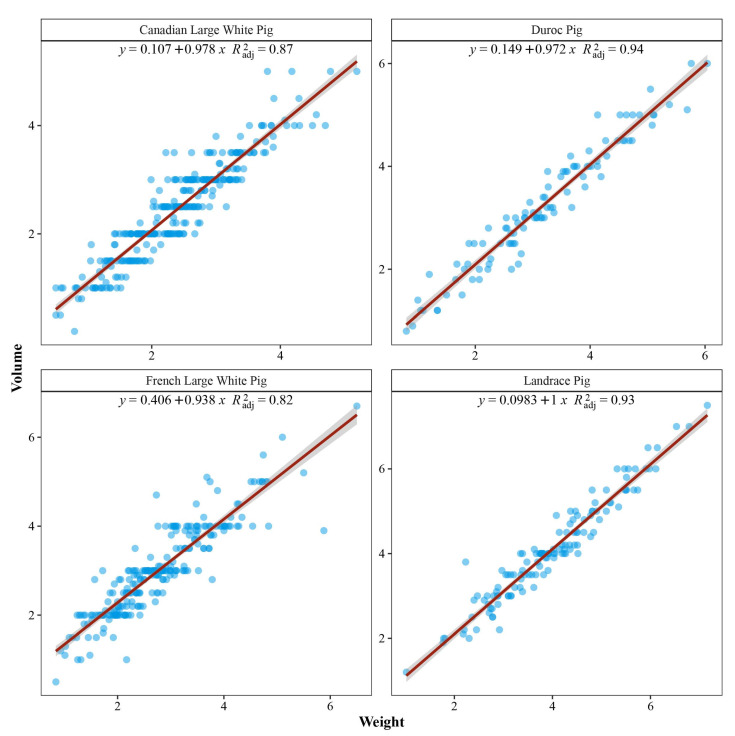
The scatterplots between the volume and weight of the right carpal gland in Canadian Large White (top-left), Duroc (top-right), French Large White (bottom-left), and Landrace (bottom-right). The red line is the fitted straight line between the volume and weight of the carpal gland.

Figure 3 shows the scatterplots between the volume and weight of right carpal glands in each population. There was a strong linear relationship between the volume and weight, and the regression coefficients were 0.98 ± 0.024, 0.97 ± 0.030, 0.94 ± 0.023 and 1.00 ± 0.027 in the four populations, respectively.

### 3.10. Relationship Between the Volume and Diverticular Number

**Figure 4:**
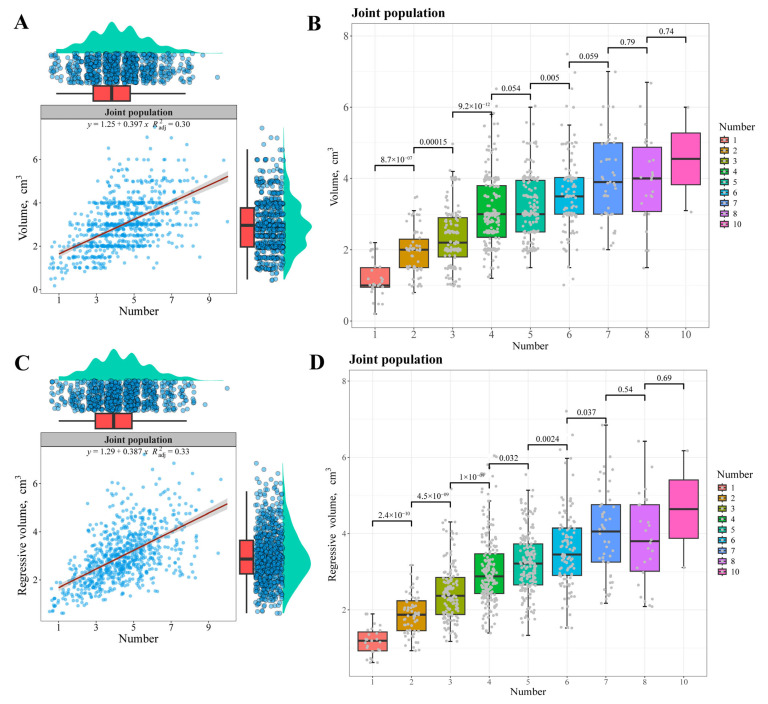
Scatter (**A**,**C**) and box (**B**,**D**) plots between the volume and diverticular number of the right carpal gland in the joint population. The red line is the fitted straight line between the diverticular number and volume of the carpal gland.

The relationship between the volume and diverticular number of the right carpal gland was explored, and both the drainage and regressive volumes had significant positive correlation with the diverticular number (Figure 4, Figures S4 and S5). The more the diverticular number was, the larger the volume (Figure 4A,C, Figures S4A and S5A). There was a strong linear relationship between them in each and joint populations, and the fit-of-goodness of each regressive model was good (*R*^2^ ≥ 0.24). The boxplots gave similar results as scatterplots, and the volumes of carpal glands were significantly different among most diverticular numbers (Figure 4B,D, Figures S4B and S5B).

### 3.11. The Effects of Carpal Gland Color on the Carpal Gland

**Figure 5:**
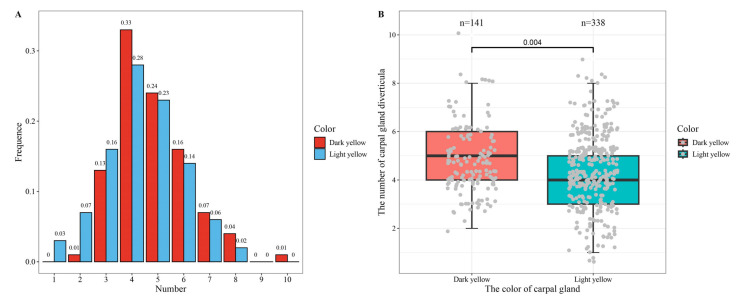
Histogram (**A**) of the diverticular number and its boxplot (**B**) against the color of carpal gland on the right foreleg in the joint population.

Figure 5 presents the boxplot and histogram of the diverticular number against the carpal gland color. A total of 479 pigs were recorded for the carpal gland color: 141 pigs with dark yellow carpal glands and 338 with light yellow carpal gland. The diverticular number of the light yellow ranged from 1 to 8, whereas the diverticular number of the dark yellow was from 2 to 10. Pigs with a dark yellow carpal gland had more diverticula than those with a light yellow carpal gland (*p* = 0.0040, Figure 5B). The color had the same effects on other traits, and the weight (*p* = 0.0003), drainage volume (*p* = 0.0003) and regression (*p* = 0.0001) volume were greater in the pigs with dark yellow carpal glands than their counterparts in the pigs with light yellow carpal glands (Figure S6).

## 4. Discussion

In this study, the diverticular number, length, width, thickness, weight, volume and color of the carpal gland were recorded in four populations, and the relationship among those traits were explored. Both population and sex had a significant effect on carpal-gland-related traits, and these traits correlated with each other positively.

In most previous studies on the carpal gland, its weight was measured postmortem after time-consuming anatomical dissection [1,9]. In this study, the diverticular number correlated strongly and positively with the weight of carpal gland (*r* ≥ 0.53, *p* < 0.01), and the larger the carpal gland was, the more the diverticula (Figure 2 and Figure S3). The diverticular number was easier to record than the other traits of the carpal gland, and it could even be counted in vivo for boars and sows. Therefore, the diverticular number could be used as a proxy for the size of the carpal gland.

In this study, the average diverticular number on each foreleg is between 4.16 and 4.71 in the four populations (Table 1 and Figure S2), and it is significantly less than that in Bama Xiang, in which the average diverticular number on each foreleg is between 5.26 and 5.54 [15]. According to previous reports [11,12], wild boars had a larger carpal gland than intensively reared domestic breed. Bama Xiang is a Chinese local breed, and it falls between wild boars and commercial breeds from an evolutionary perspective [18], so it has a larger carpal gland than Large White, Landrace and Duroc. Namely, it has more diverticula than the commercial breeds, because there are more diverticula in the large carpal glands.

Population has significant effects on all of the carpal-gland-related traits (*p* ≤ 0.01) except for the difference in diverticular numbers between the forelegs (Table 3), and this result is consistent with previous reports [15]. Northeastern Min pig had a significantly larger carpal gland than Harbin White pig, and its diverticular number was also significantly more than the latter [16]. Large White pigs from Canada and France also had significant different effects on the weight, width and volume of the carpal gland. These results indicated that not only breed but also sub-breed should be considered in the analyses of those traits.

In this study, the difference in diverticular numbers between the forelegs is not significant in each population and the joint population of four (*p* ≥ 0.32), which is consistent with our previous findings in each population (*p* ≥ 0.08). However, in our previous study, the right foreleg had more diverticula (*p* = 0.01) than the left foreleg did in the joint population of six [15], and other researchers also found that the carpal gland in the right foreleg was significantly larger (more diverticula) than that in the left foreleg [9].

The means of all traits were larger in males than in females, but only the difference in volume was significant in both the parametric and nonparametric tests (Table 4). The result was consistent with the results of many previous studies, in which the males’ carpal gland was larger and had more diverticula than the females’ [1,2,9,15].

The carpal gland was divided into dark yellow and light yellow groups according to its color, and the dark yellow had more diverticula than the light yellow (Figure 5). Previous studies also reported the carpal gland having different colors, such as brown [19], brownish-yellow [20], and pale pink or pinkish-yellow [16].

Some studies considered that the carpal gland size correlated strongly and positively with its function. The larger the carpal gland is, the stronger its function. Boars need to release more pheromones for courtship purposes during the reproductive period, so the carpal gland is larger and has more diverticula in boars than in sows [9,15]. Taking wild boars as an example, young boars leave the sow group and live alone until maturation, and they join the sow herd during the reproductive period, so they need more secretion for exchanging information [21]. In addition, wild boars have a wider acting range and spend more time roaming, and they need more secretions to mark their territory and release warning messages [22]. Therefore, the carpal gland is larger in the wild boar than commercial breeds. The size of the carpal gland varies in reproductive statuses; it is significantly larger in pregnant and lactating than in non-reproductive periods [9]. Since the dark yellow carpal gland is larger than the light yellow one, we can infer that the color of the carpal gland maybe correlates with its function.

Although the diverticular number correlated positively with the weight and volume of the carpal gland (Figure 2, Figure 4 and Figures S3–S5), there were no significant differences in weight and volume between some diverticular numbers, for example, the difference in carpal gland weight between 8 and 10 diverticular numbers (*p* = 0.69, Figure 2B) in the joint population. Due to the small sample size (only 2 pigs with 10 diverticula), the difference in weight between 8 and 10 diverticular numbers was not significant.

## 5. Conclusions

The carpal gland is a flat small stick with a length of 1.8–8.6 cm, width of 0.6–2.5 cm, and a thickness of 0.2–0.8 cm. Each carpal gland has 0 to 10 diverticula, and more than 82.3% carpal glands have 2–6 diverticula. The diverticular number of the carpal gland correlates positively with its weight and volume, and the former can be used as a proxy for the latter. The color of the carpal gland has effects on its size, and may correlate with its functions. Except for population and sex, some other factors have significant effects on the carpal gland.

## Figures and Tables

**Table 1:** Simple descriptive statistics of the carpal-gland-related traits in each population.

Trait	Population	*n*	Mean ± SD	Minimum	Maximum
Diverticular number on the left foreleg	Canadian Large White	309	4.18 ± 1.751	0	9
	French Large White	250	4.30 ± 1.337	1	8
	Landrace	130	4.62 ± 1.571	1	10
	Duroc	99	4.68 ± 1.817	1	9
Diverticular number on the right foreleg	Canadian Large White	309	4.16 ± 1.746	1	10
	French Large White	250	4.36 ± 1.364	1	8
	Landrace	130	4.68 ± 1.463	1	10
	Duroc	99	4.71 ± 1.808	1	8
Diverticular number on the both forelegs	Canadian Large White	309	8.34 ± 3.275	1	17
	French Large White	250	8.66 ± 2.504	2	16
	Landrace	130	9.31 ± 2.803	2	19
	Duroc	99	9.38 ± 3.469	2	17
The difference in diverticular numbers between the forelegs	Canadian Large White	309	0.03 ± 1.224	−4	3
	French Large White	250	−0.06 ± 1.012	−3	3
	Landrace	130	−0.06 ± 1.166	−4	3
	Duroc	99	−0.03 ± 1.054	−2	3
Weight, g	Canadian Large White	309	2.39 ± 0.848	0.51	5.19
	French Large White	250	2.75 ± 0.950	0.84	6.50
	Landrace	130	3.97 ± 1.136	1.02	7.16
	Duroc	99	3.18 ± 1.182	0.80	6.04
Length, cm	Canadian Large White	309	4.97 ± 1.112	1.8	8.2
	French Large White	250	4.89 ± 0.881	2.1	7.7
	Landrace	130	5.46 ± 0.998	2.1	7.4
	Duroc	99	4.78 ± 1.185	2.1	7.4
Width, cm	Canadian Large White	309	1.55 ± 0.305	0.6	2.3
	French Large White	250	1.73 ± 0.267	1.1	2.4
	Landrace	130	1.86 ± 0.261	1.3	2.5
	Duroc	99	1.76 ± 0.262	1.1	2.4
Thickness, cm	Canadian Large White	309	0.40 ± 0.119	0.2	0.8
	French Large White	250	0.38 ± 0.098	0.2	0.7
	Landrace	130	0.36 ± 0.092	0.2	0.6
	Duroc	99	0.37 ± 0.098	0.2	0.6
Volume, cm^3^	Canadian Large White	309	2.45 ± 0.892	0.2	5.0
	French Large White	250	2.99 ± 0.995	0.5	6.7
	Landrace	130	4.07 ± 1.179	1.2	7.5
	Duroc	99	3.24 ± 1.184	0.8	6.0
Specific gravity	Canadian Large White	305	0.988 ± 0.143	0.51	1.36
	French Large White	247	0.921 ± 0.130	0.56	1.35
	Landrace	130	0.978 ± 0.084	0.59	1.33
	Duroc	99	0.985 ± 0.108	0.63	1.32

**Table 2:** Pearson and Spearman phenotypic correlation coefficients between recorded traits.

**Trait ^1^**	**Left**	**Right**	**Difference**	**Both**	**Weight**	**Length**	**Width**	**Thickness**	**Volume**	**Specific Gravity**
Left		**0.76 ^2^**	**0.36**	**0.94**	**0.53**	**0.59**	**0.27**	**0.11**	**0.50**	*0.08*
Right	**0.74 ^3^**		**−0.34**	**0.94**	**0.57**	**0.69**	**0.29**	**0.12**	**0.55**	*0.08*
Difference	**0.34**	**−0.34**		0.01	−0.06	**−0.14**	−0.02	−0.01	*−0.08*	0.01
Both	**0.93**	**0.93**	0.0011		**0.59**	**0.68**	**0.30**	**0.12**	**0.56**	*0.08*
Weight	**0.54**	**0.58**	−0.06	**0.59**		**0.74**	**0.66**	**0.21**	**0.95**	**0.13**
Length	**0.56**	**0.66**	**−0.13**	**0.65**	**0.73**		**0.30**	0.06	**0.70**	**0.14**
Width	**0.29**	**0.30**	−0.02	**0.31**	**0.65**	**0.30**		**0.16**	**0.62**	0.01
Thickness	**0.11**	**0.11**	−0.01	**0.12**	**0.22**	*0.08*	**0.19**		**0.21**	0.03
Volume	**0.50**	**0.56**	*−0.08*	**0.56**	**0.95**	**0.68**	**0.63**	**0.22**		**−0.15**
Specific gravity	**0.09**	0.07	0.01	*0.08*	**0.16**	**0.16**	0.03	0.03	**−0.1187**	

Note: 1. Left, right and both represent the diverticular numbers on the left, right and both forelegs, respectively, and difference indicates the difference in diverticular number in the left foreleg from thatin the right foreleg. 2. The Pearson (product–moment) and Spearman (rank) phenotypic correlation coefficients are listed on the upper and lower triangles, respectively; 3. The correlation coefficients in italic and bold are significant (*p* < 0.05) and highly significant (*p* < 0.01), respectively.

**Table 3:** Least square means of the recorded traits in each population.

Trait ^1^	Canadian Large White ^2^	French Large White	Landrace	Duroc	*p* Value	
					ANOVA ^3^	Kruskal–Wallis ^4^
Left	4.20 ± 0.093 ^b^	4.31 ± 0.102 ^ab^	4.66 ± 0.143 ^a^	4.69 ± 0.163 ^a^	0.008359	0.006006
Right	4.16 ± 0.092 ^b^	4.36 ± 0.101 ^ab^	4.66 ± 0.142 ^a^	4.71 ± 0.162 ^a^	0.002745	0.001454
Difference	0.04 ± 0.065	−0.05 ± 0.072	0.00 ± 0.100	−0.03 ± 0.114	0.8038	0.7538
Both	8.36± 0.173 ^a^	8.67 ± 0.191 ^ab^	9.32 ± 0.267 ^b^	9.40 ± 0.304 ^b^	0.002205	0.001696
Weight	2.41 ± 0.057 ^d^	2.76 ± 0.063 ^c^	3.96 ± 0.088 ^a^	3.20 ± 0.100 ^b^	<2 × 10^−16^	<2.2 × 10^−16^
Length	4.99 ± 0.060 ^b^	4.88 ± 0.066 ^b^	5.45 ± 0.092 ^a^	4.80 ± 0.105 ^b^	1.007 ×10^−6^	3.043 × 10^−8^
Width	1.55 ± 0.016 ^c^	1.73 ± 0.018 ^b^	1.86 ± 0.025 ^a^	1.76 ± 0.029 ^b^	<2 × 10^−16^	<2.2 × 10^−16^
Thickness	0.40 ± 0.006 ^a^	0.38 ± 0.007 ^ab^	0.36 ± 0.009 ^b^	0.37 ± 0.011 ^ab^	0.001325	0.008112
Volume	2.46 ± 0.058 ^c^	2.97 ± 0.064 ^b^	4.07 ± 0.090 ^a^	3.28 ± 0.103 ^b^	<2 × 10^−16^	<2.2 × 10^−16^

Note: 1. Left, right and both represent the diverticular numbers on the left, right and both forelegs, respectively, and the difference indicates the difference in diverticular number on the left foreleg from that on the right foreleg; 2. The least square means with different superscript letters indicate there are significantly differences among them; 3. The *p* value is from the two-way analysis of variance (ANOVA) for population and sex, and the effect of sex is corrected; 4. The Kruskal–Wallis tests do not correct for the effect of sex.

**Table 4:** Least square means of the carpal-gland-related traits for each gender.

Trait ^1^	Male (*n* = 330)	Female (*n* = 458)	Male-Female	*p* Value	
				ANOVA ^2^	Wilcoxon Test ^3^
Left	4.52 ± 0.094	4.40 ± 0.081	0.122 ± 0.117	0.30	0.19
Right	4.50 ± 0.093	4.45 ± 0.080	0.044 ± 0.116	0.71	0.60
Difference	0.03 ± 0.066	−0.05 ± 0.056	0.078 ± 0.082	0.34	0.33
Both	9.02 ± 0.175	8.86 ± 0.150	0.165 ± 0.219	0.45	0.38
Weight	3.14 ± 0.057	3.02 ± 0.049	0.122 ± 0.072	0.09	0.04
Length	5.09 ± 0.061	4.97 ± 0.052	0.119 ± 0.076	0.12	0.04
Width	1.73 ± 0.016	1.72 ± 0.014	0.010 ± 0.021	0.64	0.21
Thickness	0.38 ± 0.006	0.37 ± 0.005	0.006 ± 0.008	0.45	0.36
Volume	3.29 ± 0.059	3.11 ± 0.051	0.176 ± 0.074	0.02	0.01

Note: 1. Left, right and both represent the diverticular numbers on the left, right and both forelegs, respectively, and difference indicates the difference in diverticular number on the left foreleg from that on the right foreleg; 2. The *p* value is from the two-way analysis of variance (ANOVA) for population and sex, and the effect of population is corrected; 3. The Wilcoxon rank sum tests do not correct the effect of population.

## Data Availability

The raw data supporting the conclusions of this article will be made available by the authors upon request.

## Supplementary Materials

The following supporting information can be downloaded at: https://www.mdpi.com/article/10.3390/ani14223231/s1, Figure S1: Schematic diagram for measuring the weight, length (L), width (W), and height (H) of the carpal gland on the right foreleg; Figure S2: The histograms of carpal gland diverticula in French Large White (A), Canadian Large White (B), Landrace (C), and Duroc (D); Figure S3: Scatter (A) and box (B) plots of the weight against the diverticular number of carpal gland on the right foreleg in each population; Figure S4: Scatter (A) and box (B) plots of the drainage volume against the diverticula number of carpal gland on the right foreleg in each population; Figure S5: Scatter (A) and box (B) plots of the regressive volume against the diverticula number of carpal gland on the right foreleg in each population; Figure S6: Boxplots of the carpal gland weight (A), drainage volume (B), and regression volume (C) against the carpal gland color; Table S1: The proportion of phenotypic variance explained by each factor and the residual.
