# Supplementary material for: Exploring the Relationship Between the Size and Diverticular Number of Carpal Gland in Four Pig (Sus Scrofa) Populations
Source: Animals (Basel). 2024 Nov 11;14(22):3231. doi: 10.3390/ani14223231 (PMC11591263; doi:10.3390/ani14223231)
Supplement: Supplementary file 1 [file animals-14-03231-s001.zip › animals-3198663-supplementary.pdf]

Supplementary data

Table S1 The proportion of phenotypic variance explained by each factor and the residual

Trait	Population	Color	Sex	Residual
Diverticular number on the left foreleg	0.0131	0.0111	0.0024	0.9734
Diverticular number on the right foreleg	0.0098	0.0068	0.0008	0.9826
Weight of carpal gland on the right foreleg	0.1835	0.0071	0.0099	0.7996
Length of carpal gland on the right foreleg	0.0691	0.0175	0.0051	0.9084
Width of carpal gland on the right foreleg	0.0362	0.0054	0.0063	0.9521
Thickness of carpal gland on the right foreleg	0.0044	0.0000	5.35E-17	0.9956
Volume of carpal gland on the right foreleg	0.1516	0.0066	0.0164	0.8254
Regressive volume of carpal gland on the right foreleg	0.1661	0.0079	0.0103	0.8157

Supplementary Figure

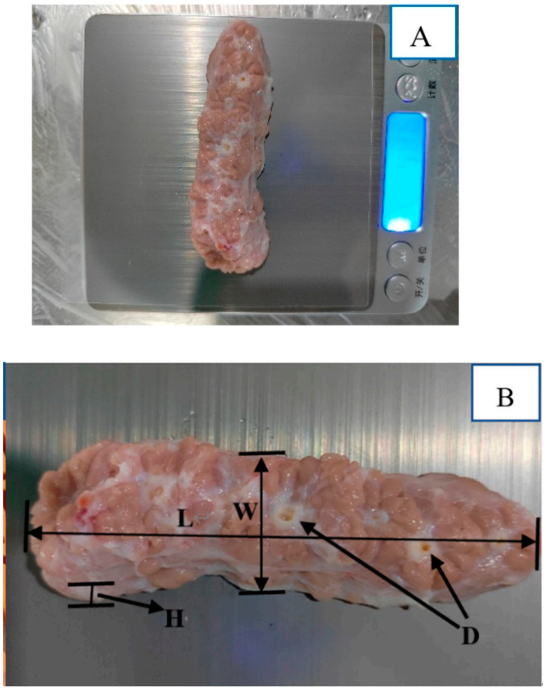

Figure S1 Schematic diagram for measuring the weight, length (L), width (W), and height (H) of the carpal gland on the right foreleg.

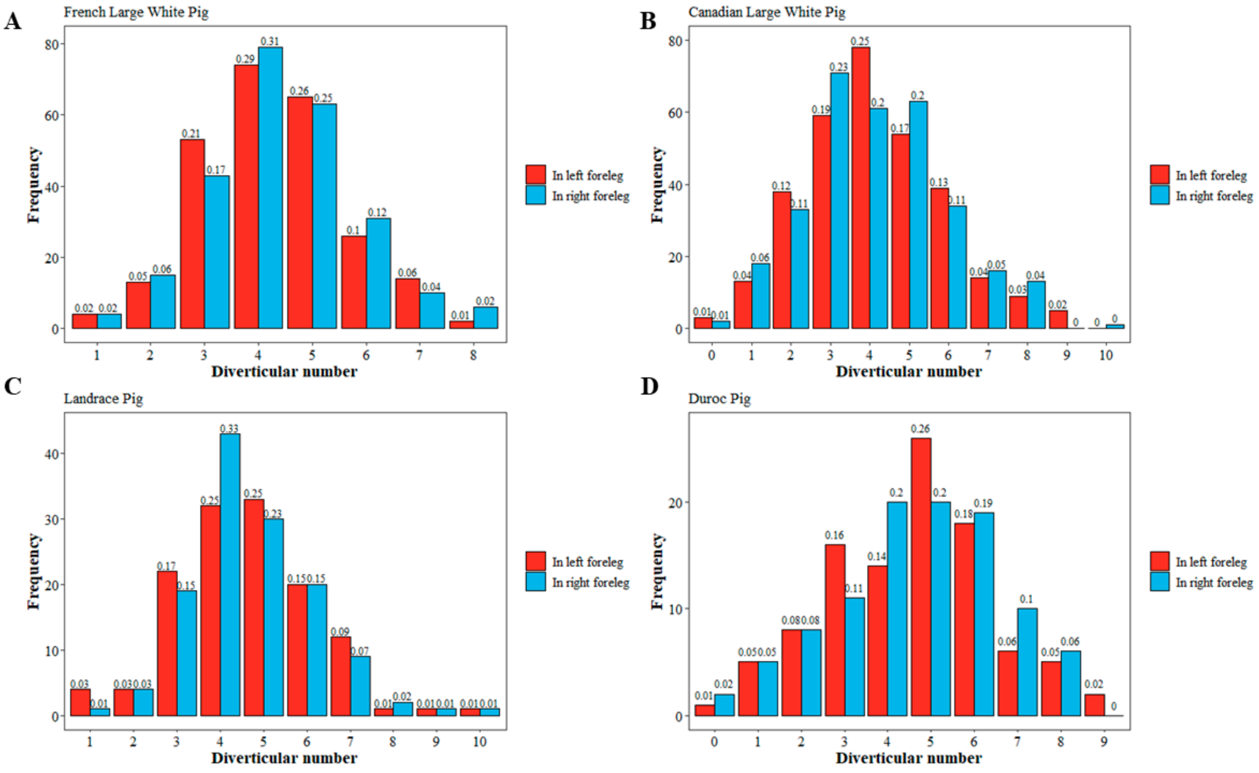

Figure S2 The histograms of carpal gland diverticula in French Large White (A), Canadian

Large White (B), Landrace (C), and Duroc (D).

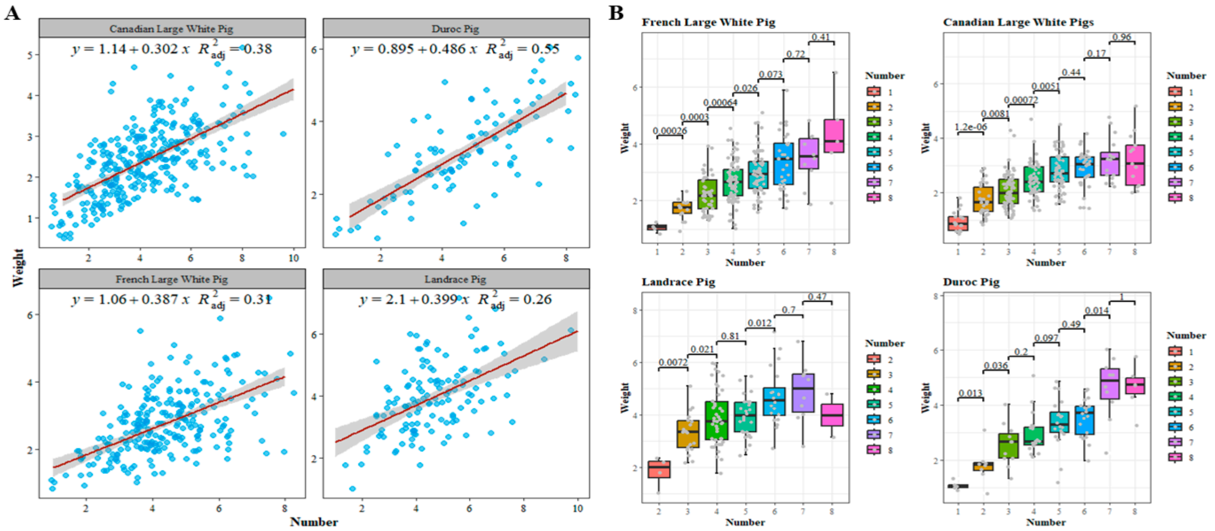

Figure S3 Scatter (A) and box (B) plots of the weight against the diverticular number of carpal gland on the right foreleg in each population.

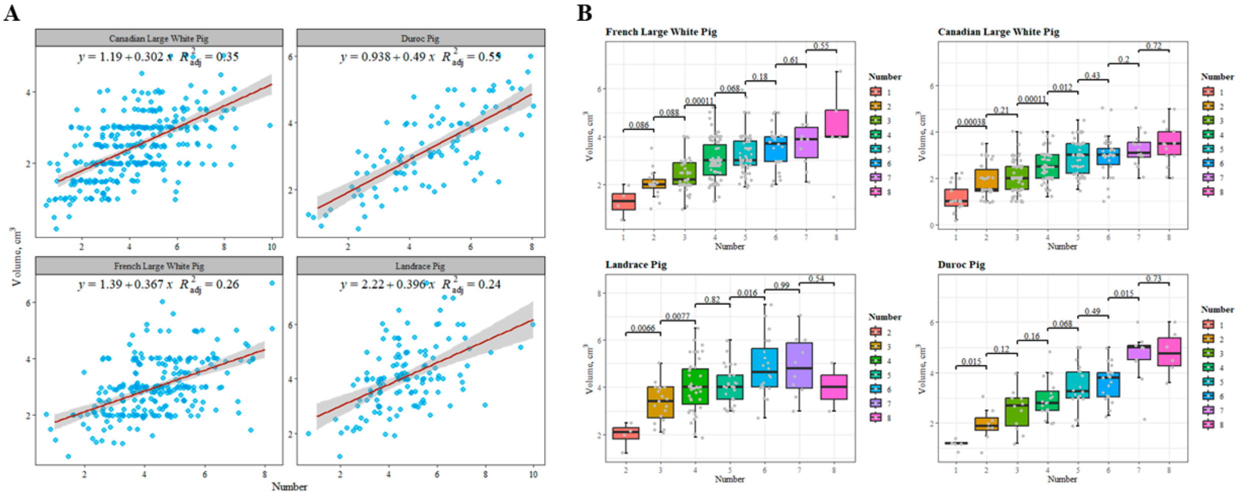

Figure S4 Scatter (A) and box (B) plots of the drainage volume against the diverticula number of carpal gland on the right foreleg in each population.

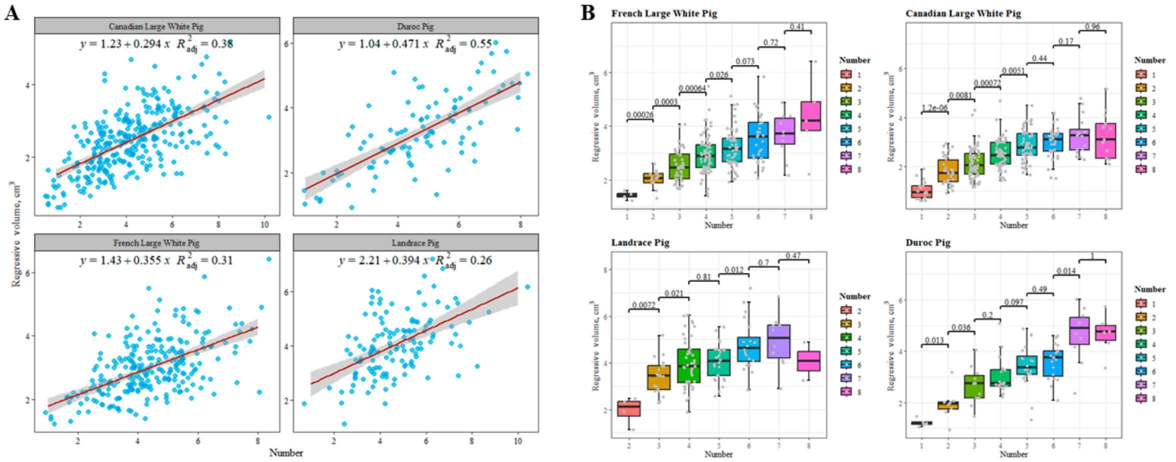

Figure S5 Scatter (A) and box (B) plots of the regressive volume against the diverticula number of carpal gland on the right foreleg in each population.

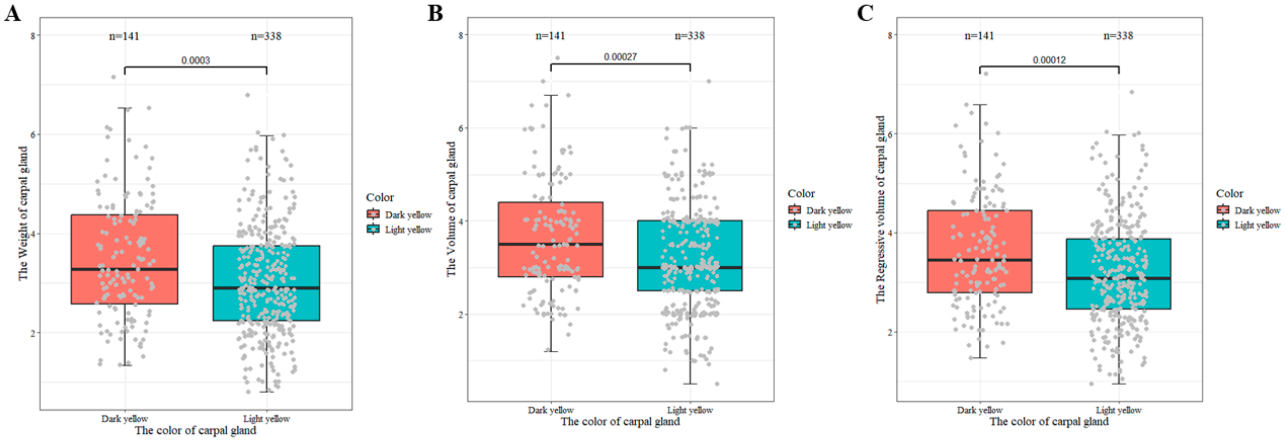

Figure S6 Boxplots of the carpal gland weight (A), drainage volume (B), and regression volume (C) against the carpal gland color.
